# Enhancement of thermoelectric efficiency of CoSb_3_-based skutterudites by double filling with K and Tl

**DOI:** 10.3389/fchem.2014.00084

**Published:** 2014-10-13

**Authors:** Ken Kurosaki, Guanghe Li, Yuji Ohishi, Hiroaki Muta, Shinsuke Yamanaka

**Affiliations:** ^1^Division of Sustainable Energy and Environmental Engineering, Graduate School of Engineering, Osaka UniversitySuita, Japan; ^2^Research Institute of Nuclear Engineering, University of FukuiTsuruga, Japan

**Keywords:** skutterudite, thermoelectric, thallium, potassium, thermal conductivity

## Abstract

The high-temperature thermoelectric properties of thallium (Tl) and potassium (K) double-filled cobalt antimonide (CoSb_3_)-based skutterudites with nominal compositions Tl_x_K_0.3_Co_4_Sb_12_ (*x* = 0.1 − 0.3) were investigated. The filling fraction of Tl in CoSb_3_ was enhanced by co-filling with K, which resulted in all of the samples showing the filled-skutterudite single phase. Owing to the high filling ratio, the carrier concentration in the sample with *x* = 0.3 was as high as 4.3 × 10^20^ cm^−3^ at room temperature. Furthermore, quite low lattice thermal conductivity (as low as 0.9 Wm^−1^K^−1^) was obtained for the sample with *x* = 0.3, probably because of strong phonon scattering by the Tl and K co-rattling effect, which resulted in a maximum *zT* of around one at 773 K.

## Introduction

Thermoelectric (TE) materials can be used for direct energy conversion from waste heat into electrical power, and have advantages of no moving parts and high reliability. The efficiency of the energy conversion of TE materials is governed by the material's dimensionless figure of merit: *zT* = *S*^2^*T*/ρκ, where *S* is the Seebeck coefficient, *T* is the absolute temperature, ρ is the electrical resistivity, and κ is the total thermal conductivity (κ = κ_lat_ +κ_el_, where κ_lat_ and κ_el_ are the lattice and electronic contributions, respectively (Ioffe, 1957; Slack, 1995; Nolas et al., 2001). Because the *zT* value directly reflects the energy conversion efficiency, development of high-*zT* materials is important for effective energy saving by recycling the waste heat by TE technology. It is considered that materials with *zT* > 1 should be obtained for practical application. To achieve such a high *zT*, a large *S*, low ρ, and low κ are required. However, *S*, ρ, and κ_el_ are strongly interrelated with each other in materials, therefore reduction of κ_lat_ is required to maximize *zT* (Ioffe, 1957; Nolas et al., 2001).

Skutterudite compounds have the composition *MX*_3_, where *M* is a transition metal, such as Co, and *X* represents a pnicogen atom, such as Sb. These compounds are body-centered cubic with 32 atoms in the unit cell and the space group *Im*3. The structure contains two voids per unit cell. When a third atom *A* is incorporated into the voids, the formula of the compounds, referred to as filled skutterudites, becomes *AM*_4_*X*_12_. The *A* atom is weakly bonded to the other atoms and “rattles,” leading to strong scattering of heat-carrying phonons. Thus, the introduction of *A* atoms into the voids of the skutterudite structure is an effective method for reducing κ_lat_. Although skutterudites filled with alkali, alkaline-earth, or rare-earth metals with *zT* > 1 have been widely reported (Morelli et al., 1997; Nolas et al., 2000; Chen et al., 2001; Lamberton et al., 2002; Puyet et al., 2004; Pei et al., 2006; Zhao et al., 2006), skutterudites filled with other elements, such as group 13 elements such as thallium (Tl), have been scarcely reported (Harnwunggmoung et al., 2010; Qiu et al., 2013; Tang et al., 2014). Recently, the vibrational frequencies of the filler atoms in cobalt antimonide (CoSb_3_)-based skutterudites have been calculated by density functional theory. It was found that the vibrational frequencies were significantly different for different chemical groups of the periodic table (Yang et al., 2007). It has been suggested that only the lattice phonons with frequencies near the vibrational frequency of the fillers can be strongly scattered via phonon resonant scattering (Shi et al., 2008). Thus, introducing filler elements belonging to different chemical groups into the cages of CoSb_3_ could introduce various distinctive filler vibrational frequencies for a broader range of lattice phonon scattering, leading to further κ_lat_ reduction (Shi et al., 2011). In the present study, we selected Tl (one of the heaviest elements) and potassium (K) (one of the lightest elements) as the double-filling combination to achieve significant reduction of κ_lat_ of CoSb_3_-based skutterudites. Based on previous studies (Pei et al., 2006; Harnwunggmoung et al., 2010) we selected the sample compositions Tl_x_K_0.3_Co_4_Sb_12_ (*x* = 0.1 − 0.3) and their high-temperature TE properties were investigated. The effect of Tl and K double-filling on the TE properties of CoSb_3_ was also investigated.

## Experimental

Polycrystalline samples of Tl and K double-filled skutterudites, Tl_x_K_0.3_Co_4_Sb_12_ (*x* = 0.1 − 0.3), were synthesized by a combination of melting, quenching, and long-term high-temperature annealing. The high-purity elements Tl (99.9%), K (99%), Co (99.99%), and Sb (99.999%) were weighed in appropriate ratios then placed in a carbon crucible in a silica tube. Considering that K rapidly evaporates at high temperatures, appropriate amounts of excess K were added to the mixtures of the starting materials. The silica tubes were sealed under vacuum, heated slowly up to 1323 K, and then quenched to room temperature. The silica tubes were then heated again up to 873 K and annealed for 1 week. The obtained ingots were crushed into powders, followed by spark plasma sintering at 923 K under a pressure of 50 MPa for 15 min in an Ar flow atmosphere.

Structural characterization was conducted using X-ray diffraction (XRD) analysis in air at room temperature with Cu Kα radiation. The microstructure and chemical composition of the samples were investigated by field emission scanning electron microscopy (FE-SEM) with energy dispersive X-ray (EDX) analysis in vacuum at room temperature. *S* and ρ were measured using a commercially-available apparatus (ULVAC, ZEM-1) in a He atmosphere. The thermal diffusivity (α) was measured by the laser flash method in a vacuum using a commercially available, thermal constant analyzer (ULVAC TC-7000). κ was evaluated via the standard equation of κ = α*C*_p_*d*, where *C*_p_ and *d* are the heat capacity and density, respectively. *C*_p_ was estimated using the Dulong–Petit model: *C*_p_ = 3*nR*, where *n* is the number of atoms per formula unit and *R* is the gas constant. All of the TE properties were measured from room temperature to 773 K.

The Hall coefficient (*R*_H_) was measured at room temperature by the van der Pauw method under vacuum with an applied magnetic field of 0.5 T. The Hall carrier concentration (*n*_*H*_) and Hall mobility (μ_H_) were calculated from *R*_H_ assuming a single band model and a Hall factor of 1, i.e., *n*_H_ = 1/(*eR*_H_) and μ_H_ = R_H_/ρ, where *e* is the elementary electric charge. The density of the bulk samples was calculated based on the samples' weight and dimensions.

## Results and discussion

**Figure 1:**
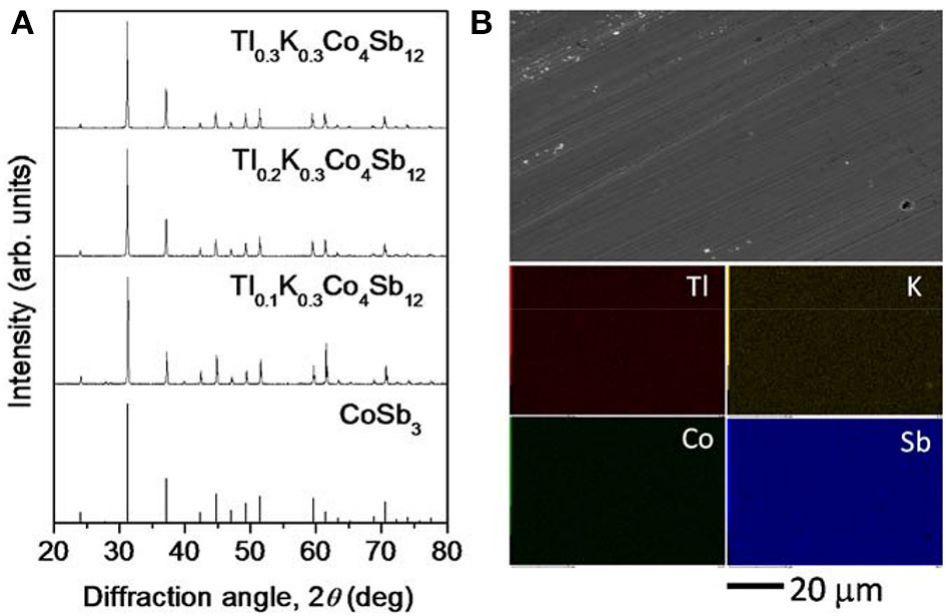
**(A)** Powder XRD patterns of polycrystalline samples of Tl_x_K_0.3_Co_4_Sb_12_ (*x* = 0.1 − 0.3), together with the peak positions of CoSb_3_. **(B)** FE-SEM and EDX mapping images of the Tl_0.3_K_0.3_Co_4_Sb_12_ bulk sample.

The powder XRD patterns of the polycrystalline samples of Tl_x_K_0.3_Co_4_Sb_12_ (*x* = 0.1 − 0.3) are shown in Figure 1A, together with the peak positions of CoSb_3_. All of the peaks in the XRD patterns were identified as peaks derived from the skutterudite phase. The lattice parameters (*a*) of the samples calculated from the XRD patterns almost linearly increased with increasing Tl content, as summarized in Table 1. The densities of the samples are summarized in Table 1. All of the samples had high densities equivalent to approximately 98% of the theoretical densities. The FE-SEM and EDX mapping images of the sample with *x* = 0.3 are shown in Figure 1B. The FE-SEM image confirmed that the sample was homogeneous. EDX analysis revealed that Tl, K, Co, and Sb were uniformly distributed on the sample surface. The chemical compositions of all of the samples determined by the quantitative EDX analysis are summarized in Table 1. The K contents in the EDX compositions were clearly lower than in the nominal compositions, probably because of the volatilization loss of K during the synthesis. The XRD and FE-SEM/EDX results revealed that all of the Tl and K added to CoSb_3_ filled the voids of the skutterudite structure, and thus all of the samples prepared in the present study were skutterudite single phases with no impurity phases.

**Table 1:** **Nominal and EDX compositions, lattice parameter (*a*), carrier concentration (*n*_H_), carrier mobility (μ_H_), and density (*d*) of the samples**.

**Nominal composition**	**EDX composition**	***a* (nm)**	***n*_H_ (10^20^ cm^−3^)**	**μ_H_ (cm^2^ V^−1^ s^−1^)**	***d* (g/cm^3^)**	***d* (%T.D.)**
Tl_0.1_K_0.3_Co_4_Sb_12_	Tl_0.1_K_0.2_Co_3.8_Sb_12.4_	0.9041 (2)	1.5	52	7.60	98
Tl_0.2_K_0.3_Co_4_Sb_12_	Tl_0.2_K_0.2_Co_3.8_Sb_12.3_	0.9059 (2)	3.0	42	7.63	98
Tl_0.3_K_0.3_Co_4_Sb_12_	Tl_0.3_K_0.2_Co_3.8_Sb_12.3_	0.9068 (2)	4.3	39	7.67	97

*The data were obtained at room temperature. Considering the uncertainty in the EDX analysis, the error bars of the EDX compositions are a maximum of 5%*.

The room temperature values of *n*_H_ and μ_H_ for the samples are summarized in Table 1. It was confirmed that increasing the Tl content increased *n*_H_. Owing to the large amounts of the filler elements Tl and K, very high *n*_H_ values (e.g., 4.3 × 10^20^ cm^−3^ for the sample with *x* = 0.3) were obtained. The μ_H_ of the samples slightly decreased with increasing *n*_H_, mainly because of the increase of carrier–carrier scattering.

**Figure 2:**
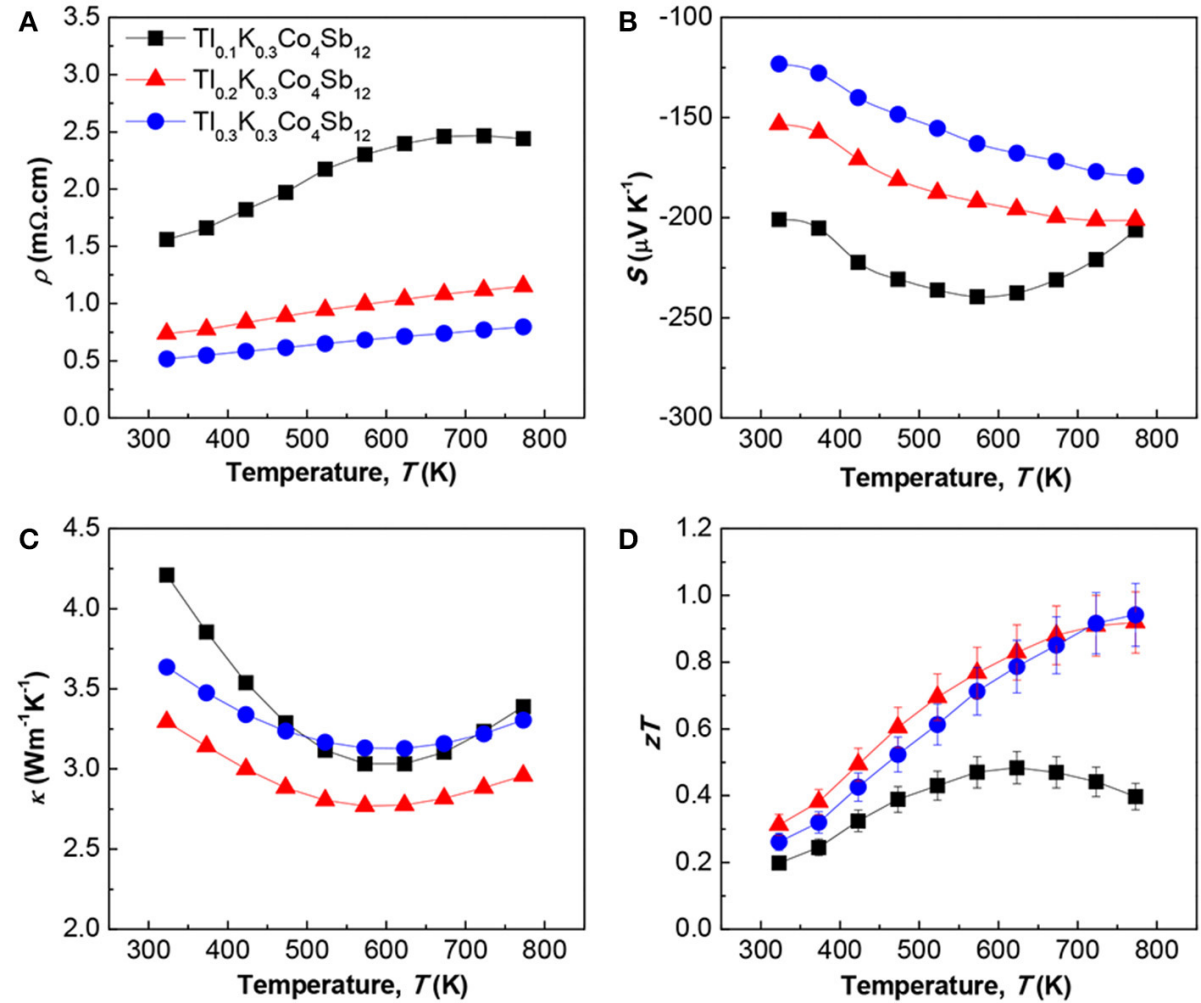
**Temperature dependences of (A) electrical resistivity ρ, (B) Seebeck coefficient *S*, (C) thermal conductivity κ, and (D) dimensionless figure of merit *zT* of polycrystalline bulk samples of Tl_x_K_0.3_Co_4_Sb_12_ (*x* = 0.1 − 0.3)**.

The temperature dependences of ρ, *S*, κ, and *zT* are shown in Figures 2A–D, respectively. As shown in Figure 2A, ρ increased with increasing temperature, showing the typical heavily-doped semiconductor behavior reported by Mallik et al. (2008). As summarized in Table 1, *n*_H_ greatly increased while μ_H_ slightly decreased with increasing Tl content, leading to a decrease in ρ with increasing Tl content. *S* was negative for all of the samples, as shown in Figure 2B, indicating that the majority of charge carriers were electrons. The absolute values of *S* decreased with increasing Tl content. The results for both ρ and *S* can be explained by *n*_H_ increasing by adding Tl. As shown in Figure 2C, all of the samples showed very low κ values. The sample with *x* = 0.3 showed higher κ than the sample with *x* = 0.2 because of the large κ_el_ of the sample with *x* = 0.3. Owing to sufficiently reduced κ, all of the samples exhibited relatively high *zT* values, as shown in Figure 2D. The maximum *zT* of around one was obtained at 773 K for the nominal compositions Tl_0.2_K_0.3_Co_4_Sb_12_ and Tl_0.3_K_0.3_Co_4_Sb_12_.

**Figure 3:**
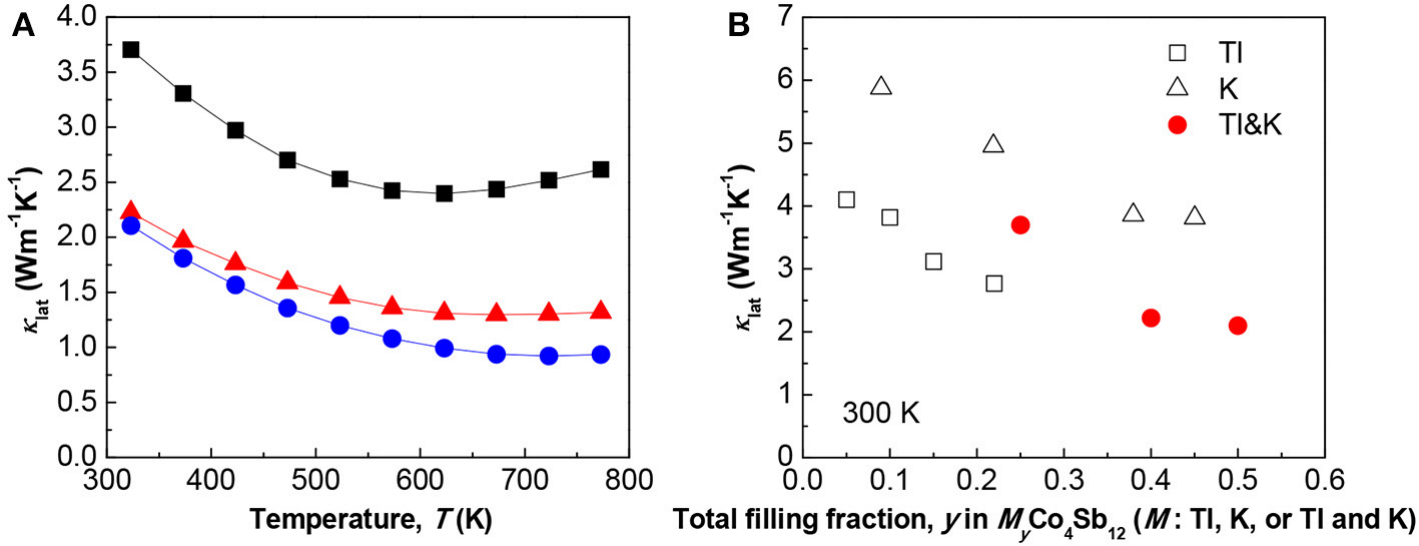
**(A)** Temperature dependence of the lattice thermal conductivity κ_lat_ of polycrystalline bulk samples of Tl_x_K_0.3_Co_4_Sb_12_ (*x* = 0.1 − 0.3). **(B)** κ_lat_ at 300 K vs. the total filling fraction *y* in *M*_y_Co_4_Sb_12_ (*M* = Tl, K, or Tl and K). The data for Tl- and K-filled skutterudites were obtained from Harnwunggmoung et al. (2010) and Pei et al. (2006), respectively.

Figure 3A shows the temperature dependence of κ_lat_ for Tl_x_K_0.3_Co_4_Sb_12_ (*x* = 0.1 − 0.3), which was obtained by subtracting the κ_el_ value from the total (measured) κ value. The value of κ_el_ can be calculated using κ_el_ = LσT, where σ is the electrical comductivity and *L* is the Lorenz number (= 2.45 × 10^−8^ W Ω K^−2^). The sample with *x* = 0.3 had the lowest κ_lat_ in the entire temperature range. Furthermore, the bipolar effect, which is observed as a rapid increase in the κ_lat_ value at high temperatures, can be seen in the sample with *x* = 0.1. It is considered that the large *n*_H_ in the samples of *x* = 0.2 and 0.3 effectively depresses the bipolar effect at high temperatures. The bipolar effect is also observed in the temperature dependence of *S* in Figure 2B, i.e., the *S* of the sample with *x* = 0.1 first decreases with temperature and then increases with temperature above about 600 K. A κ_lat_ value as low as 0.9 Wm^−1^ K^−1^ was obtained for the sample with *x* = 0.3. The κ_lat_ values obtained in the present study are relatively low compared with those of other reported filled-skutterudite compounds. These results indicate that Tl and K double-filling is an effective way to scatter heat-carrying phonons and thus achieve sufficiently low κ_lat_.

Figure 3B shows the κ_lat_ value at 300 K vs. the total filling fraction *y* in *M*_y_Co_4_Sb_12_ (*M* = Tl, K, or Tl and K). Note that, here, the filling fraction of the Tl and K double-filling system, i.e., the *y* values in (Tl, K)_y_Co_4_Sb_12_, were calculated based on the EDX compositions. In the case of the single element-filled system, it has been reported that the maximum filling limit *y* is around 0.2 (Harnwunggmoung et al., 2010) and 0.45 (Pei et al., 2006) for Tl_y_Co_4_Sb_12_ and K_y_Co_4_Sb_12_, respectively. However, in the case of the Tl and K double-filling system, the total filling fraction was as high as 50% in the voids of the skutterudite structure, in other words, *y* = 0.5 in (Tl, K)_y_Co_4_Sb_12_. This large filling fraction led to significantly reduced κ_lat_, and thus very high *zT* values around one were obtained.

## Summary

In the present study, polycrystalline samples of Tl and K double-filled skutterudites with nominal compositions Tl_x_K_0.3_Co_4_Sb_12_ (*x* = 0.1 − 0.3) were prepared and their high-temperature TE properties were investigated. This is the first attempt to co-fill group 13 elements and alkaline metals into CoSb_3_-based skutterudites. All of the samples showed the skutterudite single phase, although the maximum filling limits in the single-filled systems were *y* = 0.2 and 0.45 for Tl_y_Co_4_Sb_12_ and K_y_Co_4_Sb_12_, respectively. Owing to the large filling fraction of Tl and K, high *n*_H_ (~4.3 × 10^20^ cm^−3^) and low κ_lat_ (~0.9 W m^−1^ K^−1^) values were obtained. It can be concluded that Tl and K double-filling increases the maximum filling limit, and thus it is an effective way to reduce the κ_lat_ value of CoSb_3_. The maximum *zT* of around one was obtained at 773 K for the samples with nominal compositions Tl_0.3_K_0.3_Co_4_Sb_12_ and Tl_0.2_K_0.3_Co_4_Sb_12_.

### Conflict of interest statement

The authors declare that the research was conducted in the absence of any commercial or financial relationships that could be construed as a potential conflict of interest.

## References

[B1] Chen L. D. Kawahara T. Tang X. F. Goto T. Hirai T. Dyck J. S. (2001). Anomalous barium filling fraction and *n*-type thermoelectric performance of Ba*_y_*Co_4_Sb_12_. J. Appl. Phys. 90, 1864–1868 10.1063/1.1388162

[B2] Harnwunggmoung A. Kurosaki K. Muta H. Yamanaka S. (2010). High-temperature thermoelectric properties of thallium-filled skutterudites. Appl. Phys. Lett. 96, 202107 10.1063/1.343073921685555

[B3] Ioffe A. F. (1957). Semiconductor Thermoelements and Thermoelectric Cooling. London: Infosearch Limited

[B4] Lamberton G. A. Jr. Bhattacharya S. Littleton R. T. IV. Kaeser M. A. Tedstrom R. H. Tritt T. M. (2002). High figure of merit in Eu-filled CoSb_3_-based skutterudites. Appl. Phys. Lett. 80, 598–600 10.1063/1.1433911

[B5] Mallik R. C. Stiewe C. Karpinski G. Hassdorf R. Müller E. (2008). Thermoelectric properties of CoSb_3_ and In_0.5_CoSb_3_ skutterudite materials, in Proceedings of the 6th European Conference on Thermoelectrics (ECT) (Paris).

[B6] Morelli D. T. Meisner G. P. Chen B. Hu S. Uher C. (1997). Cerium filling and doping of cobalt triantimonide. Phys. Rev. B 56, 7376–7383 10.1103/PhysRevB.56.7376

[B7] Nolas G. S. Kaeser M. Littleton R. T. Tritt T. M. (2000). High figure of merit in partially filled ytterbium skutterudite materials. Appl. Phys. Lett. 77, 1855–1867 10.1063/1.1311597

[B8] Nolas G. S. Sharp J. Goldsmid H. J. (2001). Thermoelectrics: Basic principles and New Materials Developments. Berlin: Springer

[B9] Pei Y. Z. Chen L. D. Zhang W. Shi X. Bai S. Q. Zhao X. Y. (2006). Synthesis and thermoelectric properties of K*_y_*Co_4_Sb_12_. Appl. Phys. Lett. 89, 221107 10.1063/1.2397538

[B10] Puyet M. Lenoir B. Dauscher A. Dehmas M. Stiewe C. Müller E. (2004). High temperature transport properties of partially filled Ca*_x_*Co_4_Sb_12_ skutterudites. J. Appl. Phys. 95, 4852–4855 10.1063/1.1688463

[B11] Qiu Y. Xi L. Shi X. Qiu P. Zhang W. Chen L. (2013). Charge-compensated compound defects in Ga-containing thermoelectric skutterudites. Adv. Funct. Mater. 23, 3194–3203 10.1002/adfm.201202571

[B12] Shi X. Kong H. Li C. P. Uher C. Yang J. Salvador J. R. (2008). Low thermal conductivity and high thermoelectric figure of merit in *n*-type Ba*_x_*Yb*_y_*Co_4_Sb_12_ double-filled skutterudites. Appl. Phys. Lett. 92, 182101 10.1063/1.2920210

[B13] Shi X. Yang J. Salvador J. R. Chi M. Cho J. Y. Wang H. (2011). Multiple-filled skutterudites: high thermoelectric figure of merit through separately optimizing electrical and thermal transports. J. Am. Chem. Soc. 133, 7837–7846 10.1021/ja111199y 21524125

[B14] Slack G. A. (1995). New materials and performance limits for thermoelectric cooling, in CRC Handbook of Thermoelectrics, ed Rowe D. M. (New York, NY: CRC Press), 407–440

[B15] Tang Y. Qiu Y. Xi L. Shi X. Zhang W. Chen L. (2014). Phase diagram of In-Co-Sb system and thermoelectric properties of In-containing skutterudites. Energy Environ. Sci. 7, 812–819 10.1039/C3EE43240H

[B16] Yang J. H. Zhang W. Bai S. Q. Mei Z. Chen L. D. (2007). Dual-frequency resonant phonon scattering in Ba*_x_R_y_*Co_4_Sb_12_ (*R*=La, Ce, and Sr). Appl. Phys. Lett. 90, 192111 10.1063/1.2737422

[B17] Zhao X. Y. Shi X. Chen L. D. Zhang W. Q. Zhang W. B. Pei Y. Z. (2006). Synthesis and thermoelectric properties of Sr-filled skutterudite Sr_y_Co_4_Sb_12_. J. Appl. Phys. 99, 053711 10.1063/1.2172705

